# Identification of polymorphisms explaining a linkage signal: application to the GAW14 simulated data

**DOI:** 10.1186/1471-2156-6-S1-S88

**Published:** 2005-12-30

**Authors:** Ming-Huei Chen, Paul Van Eerdewegh, Josée Dupuis

**Affiliations:** 1 Department of Mathematics and Statistics, Boston University, Boston, MA, USA; 2 Genizon BioSciences Inc., Montreal, Canada and Department of Psychiatry, Harvard Medical School, Boston, MA, USA; 3 Department of Biostatistics, Boston University School of Public Health, Boston, MA, USA

## Abstract

We applied three approaches for the identification of polymorphisms explaining the linkage evidence to the Genetic Analysis Workshop 14 simulated data: 1) the genotype-IBD sharing test (GIST); 2) an approach suggested by Horikawa and colleagues; and 3) the homozygote sharing test (HST). These tests were compared with a family-based association test. Two linked regions with highest nonparametric linkage scores were selected to apply these methods. In the first region, Horikawa's method identified the most SNPs within the region containing the disease susceptibility locus, while HST performed best in the second region. However, Horikawa's method also had the most type I errors. These methods show potential as additional tools to complement family-based association tests for the identification of disease susceptibility variants.

## Background

Linkage analysis tends to identify broad regions of the genome that contain one or several disease susceptibility genes. However, going from a linkage peak to the actual functional polymorphisms is a daunting task. Methods that rely on linkage disequilibrium (LD), such as the transmission disequilibrium test (TDT), usually have a much better resolution for complex trait mapping. There has been recent interest in the literature for developing methods to identify polymorphisms that may be responsible for a linkage peak observed in a region. Here we apply two methods conditional on offspring genotypes [1,2] and one conditional on parental genotypes [3] to the Genetic Analysis Workshop (GAW14) simulated data for the identification of polymorphisms explaining the linkage evidence. The results are contrasted with the family-based association method implemented in TRANSMIT [4].

## Methods

To identify regions of the genome harboring susceptibility genes to Kofendred Personality Disorder (KPD), we performed nonparametric linkage (NPL) analysis, as implemented in GENEHUNTER [5], for a single replicate selected at random (replicate 71) for each population separately and for all 10 chromosomes provided. We selected the two regions with highest NPL scores (Karangar (KA) population on chromosome 9 and Danacaa (DA) population on chromosome 1), and requested the genotypes of additional single nucleotide polymorphisms (SNPs) located under these two linkage peaks. We then applied three methods, described briefly below, to identify polymorphisms that explain a linkage peak. The analyses were performed without knowledge of the true results.

### Horikawa method

To assess whether a SNP is associated with the linkage evidence, Horikawa et al. [1] suggested computing the linkage evidence in the subset of families with probands carrying the risk genotypes. They argued that affected siblings with increased identity-by-descent (IBD) sharing over what is expected by chance would be more likely to carry the risk genotypes. Hence, by selecting pedigrees based on probands carrying risk genotypes, the probability that affected siblings share two alleles IBD should increase. The significance of the change in IBD sharing in the subset of size *N*_*S *_of families with the risk genotypes is assessed using a permutation approach, by randomly selecting subsets of *N*_*S *_families, irrespective of proband genotypes.

### Genotype-IBD sharing test (GIST)

Li et al. [2] recently developed GIST to identify SNPs that can account in part for the linkage evidence in a region. They proposed a weighted analysis, in which each family is weighted according to the genotype distribution of members of the pedigree. The optimal weighting scheme depends on the model, so they suggest performing three analyses, each analysis using optimal weights for a dominant, recessive, and additive models. The maximum over all three models is used to assess whether a polymorphism partially explains the linkage evidence in a region.

### Homozygote sharing test (HST)

In contrast, Dupuis and Van Eerdewegh HST method [3] conditions on parental genotypes. They argue that if a parent is homozygous at all risk SNPs in a linked region, then it should not matter which haplotype is transmitted to affected offspring because they confer the same disease susceptibility. Hence, there should be no excess IBD sharing by affected siblings inherited from parents who are homozygous at all risk variants. However, if a particular set of SNPs is in linkage equilibrium with the susceptibility SNPs, the sharing probabilities should not depend on the parental genotypes, and the probabilities of IBD sharing from homozygous and heterozygous parents should be the same. For the intermediate situation in which the tested SNPs are in LD with risk variants, some increased sharing may be observed from homozygous parents, and the degree of excess sharing will depend on the LD between the tested SNPs and the disease SNPs. Therefore, they propose to compare the observed IBD sharing from homozygous and heterozygous parents to determine if a particular subset of SNPs explains none, some, or all of the evidence for linkage in the region. To identify whether a subset of SNPs explains some of the linkage evidence, they propose the HST statistic to test the following hypotheses H_0_: *α*_*homo *_= *α*_*het *_(>1/2) versus H_1_: 1/2 ≤ *α*_*homo *_<*α*_*het*_, where *α*_*homo *_and *α*_*het *_are the probabilities of sharing one allele IBD with respect to homozygous and heterozygous parents respectively. The HST statistic is defined as



where *N*^*homo *^and *N*^*het *^denote the number of homozygous and heterozygous parents respectively;  and  denote the number of sib pairs sharing *j* (*j*=0 or 1) allele IBD from homozygous and heterozygous parents, respectively. Because the HST is a likelihood ratio test only under genetic models in which parental transmissions are independent, we assess its significance using a permutation approach, where parental homozygosity status are randomly assigned and the original numbers of heterozygous and homozygous parents are kept constant. Once a subset of SNPs explaining some of the linkage evidence has been identified, Dupuis and Van Eerdewegh [3] test the hypotheses H_0_: 1/2 = *α*_*homo *_<*α*_*het *_versus H_1_: 1/2 <*α*_*homo *_<*α*_*het *_with the following statistic:  to determine if the subset explains all of the linkage evidence. The significance of this statistic is assessed using a permutation approach.

## Results

### Genome scan results and LD analysis

The maximum NPL scores were found on chromosome 9 in the KA population (NPL score = 5.35 at C0765) and on chromosome 1 in the DA population (NPL = 4.70 near C0052). We computed pair-wise LD measures (*D*' and *r*^2^) between markers in the two regions and found that while there was some LD on chromosome 9 (maximum *r*^2 ^= 0.89), there was little LD on chromosome 1 (maximum *r*^2 ^= 0.03).

### Single SNP analysis

**Figure 1:**
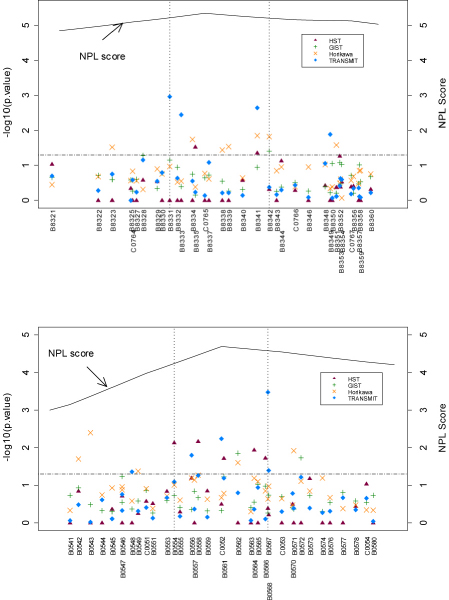
**Single SNP results for KA population on chromosome 9 (top) and DA population on chromosome 1 (bottom)**. The vertical dotted lines specify the haplotype region (HR). Significant SNPs are above the horizontal dotted line (-log_10_0.05).

Figure 1 presents the results of the single SNP analysis for the three methods (HST, GIST, Horikawa) and for TRANSMIT for chromosomes 9 (top) and 1 (bottom). For each region, 38 SNPs were tested and plotted on the *x*-axis according to map distances, while the negative of the logarithm to base 10 of the *p*-value is plotted on the *y*-axis. Table 1 presents the number of significant (*p *< 0.05) SNPs detected in the two linked regions. After consulting the answers, we defined the haplotype region (HR) to be the set of SNPs forming the haplotypes containing the disease locus.

In the KA population on chromosome 9, seven SNPs were associated (*p *< 0.05) with the linkage evidence using Horikawa et al.'s method [1], five of them within HR. In contrast, only two SNPs explained (partially) the linkage evidence using HST, both within HR, while a single SNP was identified using GIST, also within HR. TRANSMIT gave the most significant results with three SNPs (*p *< 0.01), all within HR.

In the DA population on chromosome 1, HST detected two SNPs at *p *< 0.01 (B0554, B0558) and three SNPs at 0.01 <*p *< 0.05 (B0561, B0564, B0566) that explain some of the linkage evidence, all within HR. In contrast, most of the statistically significant SNPs identified by the Horikawa's method lie outside of HR and represent type I error because there is no LD on chromosome 1. GIST identified one SNP close to the disease locus (B0562). Similar to chromosome 9, TRANSMIT yielded the most significant association with B0567, at the edge of the HR, and showed significant association with C0052 near the disease locus. SNPs significant by HST were tested to see if they explain all rather than some of the linkage evidence. None of the single SNPs explained all of the evidence for linkage on either chromosome (results not shown).

### Two-SNP analysis

**Figure 2:**
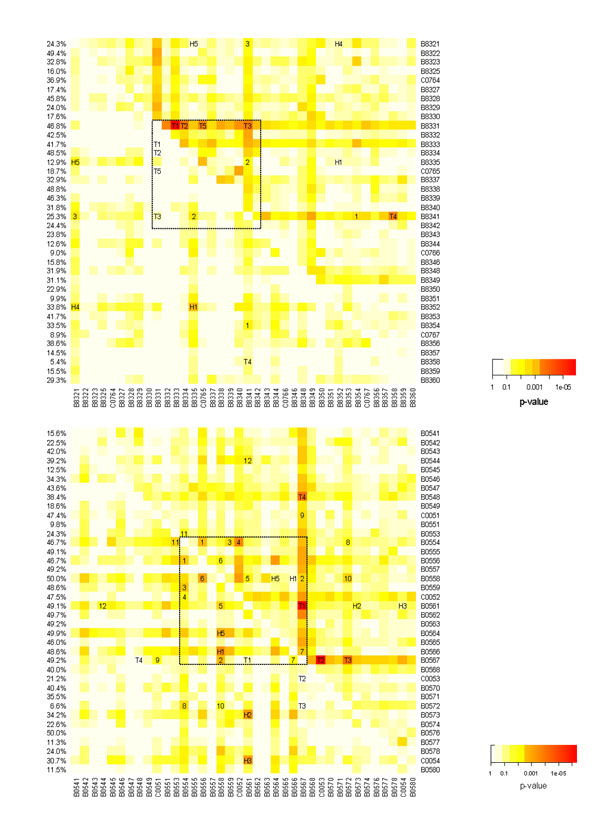
**Two-SNP results for KA population on chromosome 9 (top) and DA population on chromosome 1 (bottom). **HST *p*-values are below the diagonal; TRANSMIT *p*-values are above; estimated minor allele frequencies are on the left margin. The square identified by "i" represents the i^th ^most significant (by both HST and TRANSMIT) SNP pair ranked by the sum of the TRANSMIT and HST *p*-values. For example, in the top panel, "3" (in the first row) means that HST and TRANSMIT *p*-values for pair B8321–B8341 are both significant and the sum of their *p*-values is the third most significant. Hj and Tj represent the j^th ^significant SNP pair by HST and TRANSMIT, respectively. Note, on the top panel, "2"-H2, "3"-H3 coincide; only "2" and "3" are identified. Similarly for the bottom panel, "4"-T5, "6"-H4 coincide and only "4" and "6" are identified.

Because none of the single SNPs fully explained the linkage evidence, we looked at two-SNP combinations (SNP pairs) using HST, which generalizes easily to SNP pairs, and compared the results to TRANSMIT. Figure 2 presents the results of two-SNP analyses on chromosomes 9 (top) and 1 (bottom). The most significant single SNPs by TRANSMIT also generate significant SNP pairs with many other SNPs tested on both chromosomes.

On chromosome 9, the most significant SNP pair (B8335 and B8352, *p *= 0.003) identified by HST does not explain all of the linkage evidence, suggesting that combinations of three or more SNPs, or untyped variants, may contribute to disease susceptibility and are responsible for the linkage evidence. On chromosome 1, HST identified 32 SNP pairs explaining some linkage evidence (*p *< 0.01), with 25 of the 32 significant pairs explaining all of the linkage evidence (results not shown). The most significant SNP pair consisted of SNPs B0558 and B0566 (*p *= 0.0003), both within HR. Assessing the accuracy of prediction by either method is difficult in the absence of knowledge of the true carrier status and haplotypes of affected individuals.

The concordance of results between TRANSMIT and HST should be used in identifying interesting SNPs and SNP combinations because the two methods use complementary information in the same nuclear families. On chromosome 9, there are three significant SNP pairs by both HST and TRANSMIT, the second most significant residing within HR. On chromosome 1, there are 12 significant SNP pairs by both HST and TRANSMIT, the first 7 most significant are within HR.

## Conclusion

We applied three methods for the identification of SNPs explaining a linkage result in two linked regions in replicate 71 of the GAW14 simulated data. For single SNP analysis, on chromosome 9, Horikawa's method identified the most SNPs within the HR, while on chromosome 1, the HST method was most successful. All significant SNPs identified by HST were within HR, while Horikawa's approach appeared to generate the most type I errors. All methods pointed to some SNPs that would not have been identified by family-based association alone. Contrasting methods is a difficult task without knowledge of true carrier status at the causal SNP/haplotype.

Methods that try to explain the linkage evidence show great promise as additional tools to be used in conjunction with family-based association tests. Methods based on offspring genotypes use, in part, information already incorporated in a TDT-type test. In contrast, the HST method based on homozygosity in parents of affected individuals uses information complementary of what is used in the TDT. Future research would involve combining both TDT and HST statistics.

## Abbreviations

GAW14: Genetic Analysis Workshop 14

GIST: Genotype-IBD sharing test

HR: Haplotype region

HST: Homozygote sharing test

IBD: Identity by descent

KPD: Kofendrerd Personality Disorder

LD: Linkage disequilibrium

NPL: Nonparametric linkage

SNP: Single-nucleotide polymorphism

TDT: Transmission disequilibrium test

## Authors' contributions

JD conceived of the study and participated in its design. JD and PVE contributed to the interpretation of the data. M-HC carried out all of the analyses. All authors worked on drafting the manuscript. All authors read and approved the final manuscript.

## Figures and Tables

**Table 1:** Number of significant SNPs found in the linked regions

	No. SNPs (SNPs within HR)			
	
Region/haplotype region	HST	Horikawa	GIST	TRANSMIT
KA9 / B8331~B8342	2 (2)	7 (5)	1 (1)	4 (3)
DA1 / B0554~B0567	5 (5)	5 (1)	2 (1)	5 (3)
